# On the relationship between enamel band complexity and occlusal surface area in Equids (Mammalia, Perissodactyla)

**DOI:** 10.7717/peerj.2181

**Published:** 2016-07-06

**Authors:** Nicholas A. Famoso, Edward Byrd Davis

**Affiliations:** 1 Department of Geological Sciences, University of Oregon, Eugene, OR, United States; 2 Museum of Natural and Cultural History, University of Oregon, Eugene, OR, United States

**Keywords:** Occlusal enamel complexity, Fractal dimensionality, Equidae, PGLS, Phylogeny, Body size, Tooth area, Evolution, Phylogenetic signal

## Abstract

Enamel patterns on the occlusal surfaces of equid teeth are asserted to have tribal-level differences. The most notable example compares the Equini and Hipparionini, where Equini have higher crowned teeth with less enamel-band complexity and less total occlusal enamel than Hipparionini. Whereas previous work has successfully quantified differences in enamel band shape by dividing the length of enamel band by the square root of the occlusal surface area (Occlusal Enamel Index, OEI), it was clear that OEI only partially removes the effect of body size. Because enamel band length scales allometrically, body size still has an influence on OEI, with larger individuals having relatively longer enamel bands than smaller individuals. Fractal dimensionality (*D*) can be scaled to any level, so we have used it to quantify occlusal enamel complexity in a way that allows us to get at an accurate representation of the relationship between complexity and body size. To test the hypothesis of tribal-level complexity differences between Equini and Hipparionini, we digitally traced a sample of 98 teeth, one tooth per individual; 31 Hipparionini and 67 Equini. We restricted our sampling to the P3-M2 to reduce the effect of tooth position. After calculating the *D* of these teeth with the fractal box method which uses the number of boxes of various sizes to calculate the *D* of a line, we performed a *t*-test on the individual values of *D* for each specimen, comparing the means between the two tribes, and a phylogenetically informed generalized least squares regression (PGLS) for each tribe with occlusal surface area as the independent variable and *D* as the dependent variable. The slopes of both PGLS analyses were compared using a *t*-test to determine if the same linear relationship existed between the two tribes. The *t*-test between tribes was significant (*p* < 0.0001), suggesting different *D* populations for each lineage. The PGLS for Hipparionini was a positive but not significant (*p* = 0.4912) relationship between *D* and occlusal surface area, but the relationship for Equini was significantly negative (*p* = 0.0177). *λ* was 0 for both tests, indicating no important phylogenetic signal is present in the relationship between these two characters, thus the PGLS collapses down to a non-phylogenetic generalized least squares (GLS) model. The *t*-test comparing the slopes of the regressions was not significant, indicating that the two lineages could have the same relationship between *D* and occlusal surface area. Our results suggest that the two tribes have the same negative relationship between *D* and occlusal surface area but the Hipparionini are offset to higher values than the Equini. This offset reflects the divergence between the two lineages since their last common ancestor and may have constrained their ability to respond to environmental change over the Neogene, leading to the differential survival of the Equini.

## Introduction

Dental morphology in ungulates has been a matter of great discussion with respect to phylogeny, diet, and habitat (Simpson, 1951; Rensberger, Forsten & Fortelius, 1984; Strömberg, 2006; Heywood, 2010; Kaiser et al., 2010; Damuth & Janis, 2011). Equid dentition has been the focus of many studies as they are a modern taxon with deep phylogenetic roots and a rich fossil record of dental material (MacFadden, 1998; Famoso & Davis, 2014). A great deal of work has focused on hypsodonty (Strömberg, 2006; Mihlbachler et al., 2011) and enamel microstructure (Pfretzschner, 1993) but only recently has there been focus on quantifying occlusal enamel band complexity (Famoso, Feranec & Davis, 2013; Famoso & Davis, 2014; Famoso et al., 2016), chewing surface complexity utilizing the occlusal patch count (OPC) method (Evans & Janis, 2014), and total content of enamel quantified as a percentage of the total tooth volume (Winkler & Kaiser, 2015a; Winkler & Kaiser, 2015b).

Hipparionini and Equini are sister tribes, derived from the Merychippine-grade that lies at the base of the Equinae (MacFadden, 1998; Famoso & Davis, 2014). The Hipparionini and Equini first appear in the middle Miocene. The Hipparionini become extinct in the Pleistocene while the Equini are extant (MacFadden, 1998). Initially both tribes were present in North America and the Old World, with Hipparionini the more prevalent. For example, in the Clarendonian North American Land Mammal Age (Miocene; 12.5–9 Ma), there was a 3:1 relationship between individuals of the Hipparionini and Equini in the Great Plains region, despite similar generic diversity (Famoso & Pagnac, 2011). By the Pleistocene, Hipparionini had dwindled to only two African genera. Equini currently consists of only one genus with eight species (Orlando et al., 2009; Vilstrup et al., 2013).

Several methods have been employed to quantify complexity of the occlusal surface in mammal teeth. Indentation index, a structural density parameter that quantifies the degree of folding of the enamel pattern (Schmidt-Kittler, 1984), has been used in rodents (Schmidt-Kittler, 2002) and bovids (Gailer & Kaiser, 2014). Becerra et al. (2012) developed the enamel index which divides the length of enamel on the occlusal surface by occlusal surface area and applied it to the teeth of rodents. 3D methods, such as OPC and 3D-dental topometry, have also been employed to quantify the chewing surface topography of horse (Evans & Janis, 2014), carnivoran, rodent (Evans et al., 2007), and bovid teeth (Winkler et al., 2013). 3D methods quantify the entire occlusal surface texture that is utilized in the mastication of food stuffs as opposed to 2D methods that are focused on quantifying the relative proportion of the hardest material in the tooth, the occlusal enamel bands.

The 2D metric which has been used most recently to quantify occlusal enamel band complexity in equids, Occlusal Enamel Index (OEI), is the dimensionless ratio of the total length of enamel to the square root of the occlusal surface area of the chewing surface (Famoso, Feranec & Davis, 2013). Famoso, Feranec & Davis (2013) were unable to clearly establish whether OEI was completely independent of the confounding factor of body size scaling. Famoso, Feranec & Davis (2013) and Famoso & Davis (2014) proposed investigating other measures of complexity which were known to be independent of scaling, namely fractal dimensionality (Mandelbrot, 1983). Nonetheless, both studies identified a significant relationship of phylogeny, diet, and tooth position on enamel complexity. Famoso & Davis (2014) found major differences in enamel complexity among the four major groups of horses (“Anchitheriinae,” *Merychippus*-grade, Hipparionini, and Equini) present in the middle Miocene to Recent. Of those four, Hipparionini had the highest complexity, while Equini had the second-highest.

**Figure 1:**
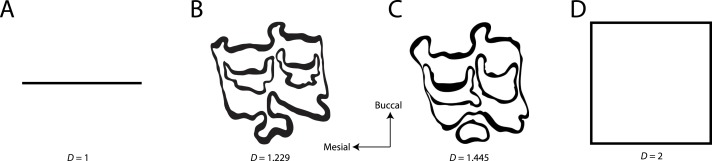
Examples of fractal dimensionality (*D*), increasing in complexity from left to right. (A) Generalized representations of a simple line; (B) Example trace of Equini P3 (MVZ 154358, *Equus asinus*); (C) Example trace of Hipparionini P3 (AMNH F:AM 71891, *Cormohipparion quinni*); (D) Generalized representations of a plane.

**Figure 2:**
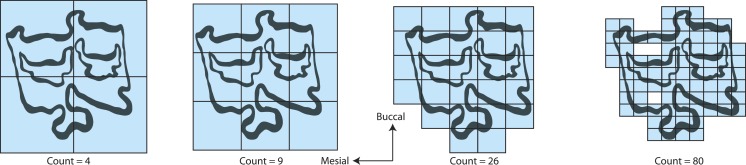
Fractal box counting method applied to a trace of the P3 of *Equus asinus* (MVZ 154358). The method uses a series of boxes (blue) of varying sizes to break down a convoluted linear pattern. The count of boxes for each box size and the box size (pixels) are then logged and a straight, fitted line is applied. The slope of this line is the inverse of the fractal dimensionality (*D*; Smith, Lange & Marks, 1996).

Fractal dimensionality (*D*) is a 2D measure of complexity, comparing the way in which detail changes with scale (Mandelbrot, 1983). Values of *D* range between 1.0 and 2.0 for a line crossing a defined area (Fig. 1). A single point has a *D* of zero; a straight line a *D* of 1, while a line so convoluted that it appears to completely cover the surface (i.e., a plane) has a *D* close to 2. An object with a *D* of 3 is a solid volume (i.e., a cube). Fractal dimensionality has been used to assign a quantitative and comparable measure of complexity to objects (e.g., leaf venation, coast lines, etc.) that cannot be conventionally measured (Theiler, 1990; Bruno et al., 2008). One efficient way of calculating *D* is the box counting method, which breaks down a convoluted linear pattern into a series of boxes with increasingly diminishing dimensions (Feder, 1988; Bruno et al., 2008). The box counting method looks at the pattern within the different boxes to investigate how the detail changes. The method is based on the number of boxes of a specific size required to fill an entire area (Fig. 2; Bruno et al., 2008). The smaller the size of the box, the more boxes are required to fill the area. The fractal dimension is calculated from the sinuosity of the line within each box. The curviness and the number of lines are used in tandem to calculate fractal dimensionality. The scalable nature of the fractal dimension will assist in removing the effects of body size from studies of tooth complexity (Gibert & Palmqvist, 1995; Famoso, Feranec & Davis, 2013).

Previously, fractal dimensionality has been used to identify taxa from suture patterns of mammalian skulls (Gibert & Palmqvist, 1995) and to investigate the evolution of suture morphology, structural functionality, and the relationship between metabolism and suture complexity in ammonites (Lutz & Boyajian, 1995; Pérez-Claros, Palmqvist & Olóriz, 2002; Pérez-Claros, 2005). Fractal dimensionality has been successfully used in quantifying the occlusal enamel band complexity of giant caviomorph rodents (Candela, Cassini & Nasif, 2013) and proboscideans (Stone & Telford, 2005) but has not yet been applied to hypsodont equids. Stone & Telford (2005) quantified enamel ridge complexity with fractal dimensionality to identify different proboscidean taxa which verified the qualitative results of Cuvier which were made over 200 years prior to their study (Cuvier, 1769; Cuvier, 1799). Candela, Cassini & Nasif (2013) focused on a single enamel crest on the lower dentition of *Eumegamys paranensis* and found that complexity is most likely related to functional stresses from the masticatory cycle. Candela, Cassini & Nasif (2013) also concluded that the dentition of *E*. *paranensis* is superficially more convoluted than that of proboscideans, a conclusion which speaks to the utility of *D* when comparing taxa of disparate body masses. Kaiser (2002) found that thin enamel band plications do not contribute to shearing of the food, but have another undetermined biomechanical function in the hipparionin equid *Cormohipparion occidentale*. Fractals have also been applied to quantifying shape and dental ecology and have been specifically applied to dental microwear using scale-sensitive fractal analysis and dental microwear texture analysis (Evans, 2013; DeSantis et al., 2013).

### Questions and hypotheses

*D* has not yet been used to quantify enamel complexity in equid dentition; therefore, we must first test whether we can achieve the same results as were found for OEI (Famoso, Feranec & Davis, 2013). To address this issue we first asked, do the Hipparionini and Equini tribes have different evolutionary trajectories responding to increased tooth abrasion over time? From previous qualitative and quantitative work, we expect the two tribes to have different levels of complexity, with Hipparionini possessing higher complexity than Equini (Quinn, 1955; Famoso & Davis, 2014). Occlusal enamel length is known to be correlated with 2D occlusal tooth area, so Famoso, Feranec & Davis (2013) developed OEI to remove that area effect; however, body size still had a measurable effect on OEI. As a consequence, the true complexity relationship between these lineages was difficult to tease apart. With *D*, a measure completely independent of area, we can now ask, does enamel band complexity increase through time in relation to increases in body size? From previous work, we expect complexity to increase through time in correlation with increasingly abrasive diets and an increase in body size (Famoso & Davis, 2014). As tooth size increases we expect there to be more space in the tooth for more complex enamel patterns because higher complexity requires enamel bands to be relatively thinner, and once enamel is too thin in absolute dimensions, it will wear enough that it will no longer effectively protrude above the dentin (Kaiser, 2002). Consequently, larger teeth can support more complexity with enamel bands above this threshold. We also expect there to be a different relationship between the two tribes, with Hipparionini being more complex.

## Methods

We took scaled (centimeter), oriented digital photographs of the occlusal surface of fossil and modern equid dentitions. Photographs were taken with a Kodak DC290 (1,792 ×1,200 pixels; 72 dpi) and Olympus Stylus Tough (3,648 × 2,736 pixels; 314 dpi) cameras. We selected specimens which were in medial stages of wear (no deciduous premolars or teeth in extreme late stages of wear). Skulls and complete to nearly complete tooth rows were preferred because we can be more confident in taxonomic identification and tooth position. Isolated teeth were also included when more complete tooth-rows were not available for a taxon. Original photos were saved as jpegs.

**Figure 3:**
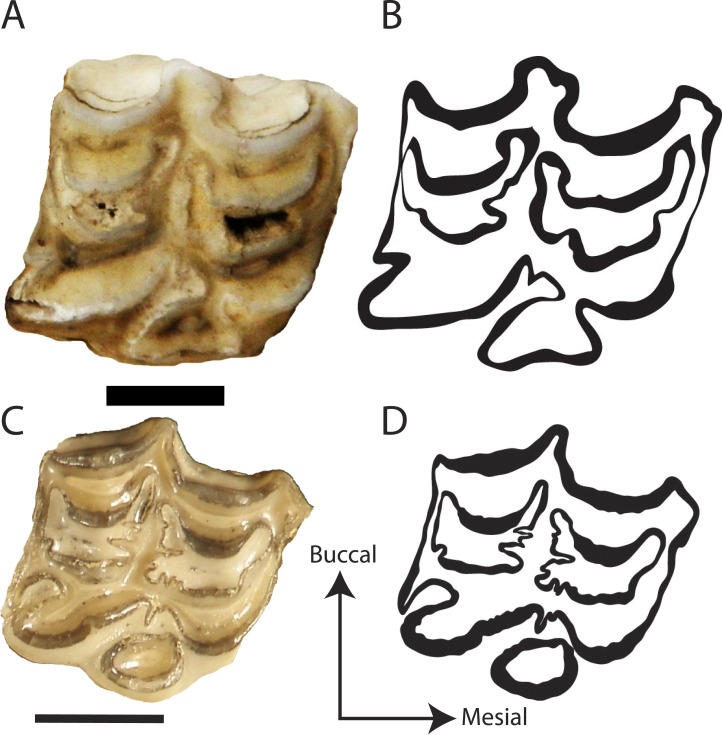
Representative photos and traces of Hipparionini and Equini taxa. (A) Photograph of the P3 of the Equini *Equus caballus* (UOMNH B-9092); (B) Trace of the P3 of the Equini *Equus caballus* (UOMNH B-9092); (C) Photograph of the P3 of the Hipparionini “*Neohipparion*” * republicanus* (UNSM 84000); (D) Trace of the P3 of the Hipparionini “*Neohipparion*”* republicanus* (UNSM 84000). Scale bars equal 1 cm.

We digitally traced 98 teeth by hand using a mouse and a Wacom Graphire3 USB tablet on original jpeg images in Adobe^®^ Illustrator^®^ CS5 version 15.0.2 producing TIFF files for fractal analysis. Only one tooth per individual was traced (Fig. 3). The preference was for P3, but if not available the next complete tooth between P3-M2 was measured. The sample comprised 31 Hipparionini and 67 Equini teeth representing 35 species (Table 1). Famoso & Davis (2014) found that all teeth in the tooth row, with the exception of the P2 and M3, had statistically similar occlusal enamel band complexity for equids. As a result, we restricted this analysis to the P3-M2 to reduce any effects from tooth position on the analysis; however, a majority of the specimens were P3s (Supplemental Information 1).

**Table 1:** Summary of species analyzed in this study and species averaged fractal dimensionality (*D*) and occlusal tooth area (cm^2^).

Tribe	Genus and species	*n* (*D*)	Mean (*D*)	SD (*D*)	*n* (area)	Mean (area)	SD (area)
Equini	*Calippus placidus*	2	1.5180	0.0891	2	2.0815	0.0629
Equini	*Calippus* sp.	1	1.3580	NA	1	3.6030	NA
Equini	*Equus asinus*	1	1.2290	NA	1	5.1590	NA
Equini	*Equus caballus*	4	1.4373	0.0549	4	7.5205	1.6367
Equini	*Equus calobutus*	4	1.3223	0.0714	4	7.2530	1.9051
Equini	*Equus complicatus*	1	1.4050	NA	1	6.0810	NA
Equini	*Equus conversidens*	1	1.3270	NA	1	5.3290	NA
Equini	*Equus excelsus*	4	1.3810	0.0879	3	6.6210	1.1453
Equini	*Equus fraternus*	1	1.3030	NA	1	8.7110	NA
Equini	*Equus grevyi*	1	1.2760	NA	1	7.5950	NA
Equini	*Equus idahoensis*	6	1.3293	0.0418	5	6.5108	0.7770
Equini	*Equus occidentalis*	1	1.4240	NA	1	8.2630	NA
Equini	*Equus quagga*	4	1.2483	0.0176	4	5.6980	0.4205
Equini	*Equus scotti*	7	1.3453	0.0598	7	6.8604	1.7839
Equini	*Equus simplicidens*	13	1.3949	0.0330	9	7.8007	1.6641
Equini	*Equus* sp.	5	1.3266	0.0221	5	5.2488	0.5808
Equini	*Equus* spp.	4	1.3075	0.0076	4	6.0648	0.5734
Equini	*Pliohippus mirabilis*	1	1.4010	NA	0	NA	NA
Equini	*Pliohippus pernix*	1	1.4000	NA	1	3.8970	NA
Equini	*Pliohippus* sp.	1	1.3980	NA	1	5.2170	NA
Equini	*Protohippus perditus*	2	1.3870	0.0170	2	3.3080	0.0198
Equini	*Protohippus* sp.	2	1.3880	0.0523	2	3.0135	0.5211
Hipparionini	*Cormohipparion goorisi*	1	1.4150	NA	1	3.8000	NA
Hipparionini	*Cormohipparion ingenuus*	1	1.3170	NA	1	3.1690	NA
Hipparionini	*Cormohipparion occidentale*	4	1.3493	0.0326	4	4.3823	1.3176
Hipparionini	*Cormohipparion quinni*	1	1.4450	NA	1	4.5870	NA
Hipparionini	“*Cormohipparion*”* sphenodus*	1	1.4270	NA	1	5.3150	NA
Hipparionini	*Neohipparion affine*	5	1.4334	0.0821	5	3.6038	0.9205
Hipparionini	*Neohipparion eurystyle*	2	1.3520	0.0764	2	4.0775	0.0700
Hipparionini	*Neohipparion leptode*	1	1.4200	NA	1	4.2290	NA
Hipparionini	“*Neohipparion*”* republicanus*	3	1.4593	0.0577	3	3.4333	0.4258
Hipparionini	*Neohipparion* sp.	5	1.4622	0.0905	5	3.9066	0.7120
Hipparionini	*Pseudhipparion gratum*	2	1.3715	0.0445	0	NA	NA
Hipparionini	*Pseudhipparion* sp.	4	1.4600	0.1061	4	3.3113	0.3720
Hipparionini	gen. *et* sp. indet.	1	1.4850	NA	1	3.0090	NA

**Notes.**

NA no value *n* number of specimens SD standard deviation

We calculated *D* on the traces using the fractal box count method in the ij.plugin.filter package (class FractalBoxCounter) in the NIH image analysis program ImageJ version 1.45 for Windows (http://rsb.info.nih.gov/ij/). The fractal box counting method in ImageJ counts the number of boxes of a given size needed to cover a binary border that is one pixel wide and is repeated for boxes that are 2–64 pixels wide (Smith, Lange & Marks, 1996). A straight, fitted line is then applied to the log of size (pixel width) versus the log of the box count and the slope of that line is the inverse of the fractal dimension, *D* (Smith, Lange & Marks, 1996). The box counting method only looks at the exterior edges of the occlusal enamel bands so occlusal enamel band thickness is not considered with this method and is not considered to influence *D*. True occlusal surface area of the tooth was collected from Famoso & Davis (2014). True occlusal surface area is defined as the two-dimensional area constructed as a polygon following the outer edge of the occlusal surface, including any cementum that may exist outside of the enamel, where cementum on the lingual side is part of the occlusal surface while that on the buccal is not (Famoso & Davis, 2014).

**Figure 4:**
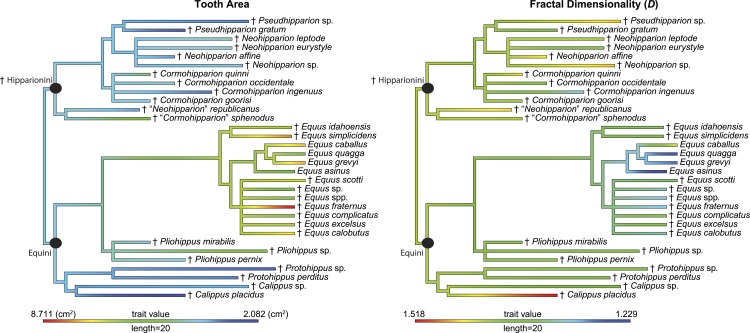
Phylogeny used in this study with continuous characters, tooth area and fractal dimensionality (*D*), mapped onto the tree. This tree is a time-scaled (Ma) informal supertree. Characters were mapped using the contMap function in the phytools package version 0.4–45 (Revell, 2012) implemented in R. Length refers to the length of the legend in units of branch length. † = extinct taxon.

Morphological characters cannot be considered statistically independent among evolutionarily related taxa (Felsenstein, 1984; Harvey & Pagel, 1991), therefore we employed methods to account for phylogenetic relatedness. A Shapiro–Wilk W test of *D* values revealed the data for the Hipparionini and Equinini tribes to be normally distributed and a Bartlett test of *D* for the two tribes indicates equal variances. We then ran a *t*-test on *D* values of specimens between the two tribes. To directly look at the relationship between occlusal surface area and *D* within a phylogenetic context, we performed a phylogenetic generalized least squares regression (PGLS; Grafen, 1989) using Pagel’s *λ* (Pagel, 1997) to incorporate the estimated phylogenetic covariance structure from the regression, as implemented in the caper package version 0.5 in R version 3.0.2 (Orme et al., 2011; R Core Team, 2013). PGLS requires species averages for each continuous variable used in the analysis (Grafen, 1989). Pagel’s *λ* is a measure of the phylogenetic signal of the residuals from the regression (Revell, 2010), or the degree to which the variance in the residuals between species can be explained by the phylogeny. If no phylogenetic signal is present (*λ* = 0), the PGLS collapses back to a generalized least squares model (GLS), allowing the use of standard parametric statistical methods. We used an informal supertree derived from the congruent phylogenies of MacFadden (1998), Kelly (1998), Hulbert (1993), and Orlando et al. (2009) (Fig. 4; Supplemental Information 2). The supertree has zero length branches, therefore we time calibrated the tree using the timePaleoPhy function with type = “zelba” to account for zero length branches, vartime = 1, and add.term = T using the paleotree package version 1.8.2 (Bapst, 2012) in R version 3.0.2 (R Core Team, 2013). All other arguments in timePaleoPhy are default. The first and last occurrences used to time calibrate the tree were collected from the Paleobiology Database (http://paleobiodb.org/) on September 9, 2015 (Supplemental Information 3). Analyses were run on the two tribes independently of one another and on a unified tree of the tribes. Raw data and R code are presented in Supplemental Information 1 and 4.

**Table 2:** *t*-test results for fractal dimensionality (*D*) versus Tribe-level affiliations.

*t*-value	Degrees of freedom	*p*-value	Equini mean	Hipparionini mean
–4.502	62.073	<0.0001	1.359	1.430

## Results

**Figure 5:**
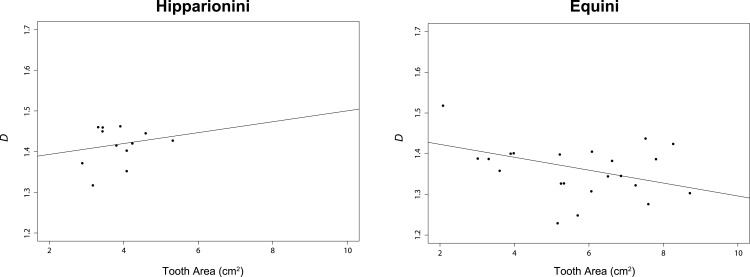
Results of our phylogenetic generalized least square regression (PGLS) for fractal dimentionality (*D*) and occlusal tooth area (cm^2^) for the Equid tribes Hipparionini and Equini. The *p*-value for the Hipparionini regression is not significant.

For this study, we assumed *α* = 0.05. The *t*-test comparing *D* between both tribes was significant (*p* < 0.0001, (Table 2)). The PGLS for Hipparionini yielded a slightly positive but non-significant relationship between *D* and occlusal surface area (*p* = 0.4912), while the PGLS for Equini produced a negative, significant relationship for these two variables (*p* = 0.0176) (Table 3 and Fig. 5). The PGLS for a single, unified tree of both Hipparionini and Equini yielded a negative, significant relationship for these two variables (*p* = 0.0040) (Table 3). The *t*-test comparing the slopes of the Hipparionini and Equini PGLS was not significant. Pagel’s *λ* for the analysis of *D* and occlusal surface area together indicated no phylogenetic signal in either tribe or both tribes when analyzed in a unified tree (Table 4). However, each character has phylogenetic signal for Equini and the unified tree when they are independently tested (Table 4). Because the analysis returned NA values for the occlusal surface area of Hipparionini, the sample size must be too small to calculate phylogenetic signal for either character (Table 4).

**Table 3:** Summary statistics for three PGLS regressions for fractal dimensionality (*D*) versus occlusal surface area.

	*λ*	Lower 95% CI	Upper 95% CI	Slope	Standard error	*t*-value	*p*-value	Multiple *R*^2^	Adjusted *R*^2^
Hipparionini	0	NA	NA	0.0134	0.0187	0.7146	0.4912	0.0486	–0.0466
Equini	0	NA	0.935	–0.0159	0.0071	–2.2299	0.0176	0.1991	0.1591
Equinae	0	NA	0.832	–0.0196	0.0063	–3.1209	0.0040	0.2451	0.2199

**Notes.**

NA no value CI confidence interval

**Table 4:** Phylogenetic signal in fractal dimensionality (*D*) and occlusal tooth area for the equid tribes Hipparionini and Equini.

	Hipparionini *D*	Hipparionini area	Equini *D*	Equini area	Equinae *D*	Equinae area
*λ*	0.000	NA	0.505	0.800	0.462	0.747
Upper 95% CI	NA	NA	0.960	NA	0.886	0.995
Lower 95% CI	NA	NA	NA	0.389	0.080	0.389

**Notes.**

NA no value CI confidence interval

## Discussion

Using the method of fractal dimensionality, we have been able to corroborate previous work using other measures of complexity in equids (Famoso & Davis, 2014), showing Hipparionini have significantly higher complexity than equines. Our PGLS results and *t*-test of the two slopes indicate a negative relationship between occlusal surface area and *D* for the Equini, and an equivocal relationship for the Hipparionini. We cannot reject the hypothesis that the Hipparionini have the same slope as the Equini, but the data are so scattered that we are circumspect in our interpretation of them. At least for Equini, as the chewing surfaces of their teeth become larger, *D* decreases. This correlation is intriguing, because previous work has shown a positive correlation between occlusal enamel length and occlusal surface area of the tooth in all equids (Famoso, Feranec & Davis, 2013), which suggested an increase in occlusal enamel band complexity with body size. Thus, we can see that the body-size scaling of the occlusal enamel band length was obfuscating the underlying pattern of occlusal enamel band complexity: future studies need to use scale-independent measures of complexity like *D* to avoid this problem. The similar pattern of decreased occlusal enamel band length with increased tooth area is observed in the bovid genus *Myotragus* (Winkler et al., 2013), suggesting that the relationship between occlusal enamel band morphology and tooth area may reflect selective pressure on increased efficiency in food processing with larger body mass operating across ungulates and not simply in equines. Alternatively, it is possible that the relationship between these two variables is controlled by developmental forces, because the dental wear properties of the tooth are mediated by the action of enamel organs during tooth development. A negative correlation between tooth area and occlusal enamel band complexity could be caused by, for example, an upper limit on the volume-filling convolutions possible in enamel organ growth and enamel/dentine production. If the observed pattern is governed by constraint in tooth development, it could be possible that the complexity-area relationship would change with wear state, because the wear states sample different stages of tooth ontogeny. Because we carefully controlled the wear stage of the specimens included in this analysis, we cannot address that possibility here. It would be informative to follow up with a longitudinal study of a single modern population or sample individuals from all age groups in a single fossil population and study whether this occlusal enamel band complexity-area relationship holds through a lifetime of tooth wear.

Additionally, while both occlusal surface area and *D* independently have phylogenetic signal, there is no phylogenetic signal in the residuals of the regressions of occlusal surface area and *D*, suggesting that the correlation between the two characters is not caused by evolutionary constraint. Consequently, the strong relationship between the two may reflect a functional or developmental connection between *D* and occlusal surface area that overrides any history of shared relationships. Both characters possess phylogenetic signal when considered on their own, indicating that these lineages do somewhat constrain values of occlusal surface area and *D*. The characters have phylogenetic signal alone but no signal together, suggesting selection strong enough to override inherited values of the characters.

The correlational nature of the PGLS does not allow us to speculate on which trait might be driving the other. One could make the best argument for selection on body size (and, consequently, occlusal surface area), which would then drive enamel band complexity as a spandrel (*sensu* Gould & Lewontin, 1979) if developmental constraints are controlling the observed relationship. The alternative would be that selection on or constraint of complexity shaped body size distributions in these species.

It is difficult to construct a scenario where selection on occlusal enamel band complexity drives body size if the two characters are negatively correlated. Famoso, Feranec & Davis (2013) showed a positive correlation between enamel band complexity (measured using OEI) and increased grazing diet in extinct and extant ungulates. If changing diets were the primary driver of horse evolution from the Miocene to Recent, body size change could be a spandrel of selection (*sensu* Gould & Lewontin, 1979) on optimal occlusal enamel band complexity. Because we have not yet explored the relationship between diet category and occlusal enamel band complexity measured using *D*, we cannot sensibly speculate on the relationship here.

Horses were transitioning from a browsing to a grazing diet through the Miocene to Recent (MacFadden, 2005), but most work has assumed that with opening habitats, large body size was selected for optimal foraging across large distances (e.g., Janis, 1993; Smith et al., 2010; Mihlbachler et al., 2011). If this were so, one would expect the selection on body size to have driven a decrease in occlusal enamel band complexity over time. Future work will need to parse out the relative roles of changes in body size and diet in the evolution of resistance to tooth wear. As a first step, it would be worth investigating the complexity-diet relationship in horses using *D* once proper dietary reconstructions can be calculated for an appropriate sample of taxa. As dietary reconstructions have only been calculated on a small number of the taxa sampled in this study, we were unable to address the relationship among body size, diet, and occlusal enamel band complexity measured using *D*.

Hipparionini and Equini do not have a significantly different relationship between occlusal enamel band complexity and occlusal surface area, but the line of the Hipparionini regression is shifted upward: this lineage features consistently more complex occlusal enamel bands, a qualitative difference first noted by Quinn (1955) when he erected the two tribes. As such, the Hipparionini and Equini express distinct dental morphological solutions, with hipparionines producing higher enamel band complexity and equines producing teeth with greater hyposodonty. In this way, the two clades were able to accommodate the dental wear induced by foraging in open habitats of both the New and Old Worlds (MacFadden, Solounias & Cerling, 1999; Passey et al., 2002; Maguire & Stigall, 2008; Uno et al., 2011; Famoso & Davis, 2014; Loffredo & Desantis, 2014).

When the two tribes are analyzed together, the overall relationship between occlusal enamel band complexity and tooth area (Table 3) places the Hipparionini and Equini on a single linear trend, with the Hipparionini occupying a low tooth area/high occlusal enamel complexity space that fits onto the left end of the Equini distribution (compare the two panels of Fig. 5). This fit between the two distributions suggests that Equini are simply larger than Hipparionini, but on the same trend, and as a result their complexity is lower. Phylogenetic signal can be invoked to explain why the Hipparionini tribe has a relatively lower crowned tooth and higher occlusal enamel band complexity and the Equini tribe has a relatively higher crowned tooth and lower occlusal enamel band complexity (Quinn, 1955; Famoso & Davis, 2014). That is, when the two groups originated, the common ancestor of the Hipparionini possessed a smaller body-size/higher occlusal enamel complexity than the common ancestor of the Equini, and the two groups retained those differences as their body size and occlusal enamel complexity evolved. Although Evans & Janis (2014) found similar three dimensional chewing surface complexity between both tribes, this metric is the result of wear more so than enamel band complexity, pointing to a different aspect of evolving ecology. The processes behind the eventual extinction of the Hipparionini and persistence of the Equini are currently unclear, but our investigation of the differences in their occupation of enamel band complexity space provides an important insight. Maguire & Stigall (2009) found that niche partitioning and the fluctuation of niches across North America were a factor affecting the relative success of these two tribes over time, suggesting that climate change may have been a major driver in the dynamic relative abundance of the two tribes and the extinction of Hiparionini.

Fractal dimensionality has shown great utility for investigating tooth enamel band complexity (Stone & Telford, 2005; Candela, Cassini & Nasif, 2013), but has promise for several other lines of investigation. This tool alleviates the effects of allometric scaling, allowing a more nuanced investigation of the evolution of enamel band complexity in any setting (e.g., Gibert & Palmqvist, 1995; Stone & Telford, 2005; Candela, Cassini & Nasif, 2013). Other potential applications include applying *D* to the lateral profiles of carnivore dentition in the context of feeding ecology and evolutionary relationships, or to quantify the overall complexity of an entire community of herbivores. Crabeater seals (*Lobodon carcinophaga*) have a complex lateral dental profile as a response to feeding on Antarctic krill (Adams, 2005), and would have higher *D* values than other Antarctic marine carnivorans (e.g., leopard seals (*Hydrurga leptonyx*)). An analysis of *D* would be complementary to other analyses which used OPC to infer dietary ecology of carnivorans from the occlusal surface topography (Evans et al., 2007).

Applying *D* to an entire mammalian herbivore community could allow one to investigate the relationship between diet and occlusal enamel band complexity between sites and to potentially tease out niche partitioning within a site or group of sites. For instance, communities of herbivores with higher *D* values would be expected in localities where microwear and isotopic analysis predict grazing diets. Within a community, browsing taxa would be expected to have a lower enamel band complexity than grazing taxa, allowing for a first order assessment of diet. An average *D* value for each site could be calculated and then compared, or *D* could be calculated for each species in a site and compared.

In the end, we can show that for the hypsodont equids in our study, occlusal enamel complexity and tooth size are negatively related. Additionally, because the slopes of the PGLS for Hipparionini and Equini were not significantly different from one another, we cannot reject the hypothesis that Hipparionini and Equini have a similar negative relationship between occlusal enamel complexity and occlusal surface area. Hipparionini show a positive relationship between these two values, but the effect size is small, the uncertainty is large (Table 3), and the combined analysis of the two tribes places them on a single linear trend, so there is a chance that a larger sample size would cause the Hipparionini trend to converge on the more robust slope estimated for Equini. Despite the equivocal results comparing their slopes, occlusal enamel band complexity values for Hipparionini are significantly higher than those for Equini. When both tribes are analyzed together, Hipparionini are on the same linear trend as Equini, suggesting that Equini simply have larger tooth areas, but the two clades are controlled by the same relationship between tooth area and occlusal enamel band complexity. The size difference between the two tribes, then, can entirely explain their difference in occlusal enamel band complexity. This difference in complexity reflects divergence between the two lineages since their last common ancestor and suggests the two tribes differentiated by exploring different parts of the ecomorphospace, with Hipparionini maintaining smaller size, more complex occlusal enamel banding, and lower hypsodonty, while the Equini maintained larger size, less complex occlusal enamel banding, and higher hypsodonty (hypsodonty explored in Famoso et al., 2016). There is strong phylogenetic signal for both *D* and occlusal surface area independently; however, there is no phylogenetic signal for the relationship between *D* and occlusal surface area. Equids have a strong correlation between occlusal surface area and enamel band complexity, and tribe-level differences in this relationship may have constrained their ability to respond to environmental change over the Neogene, leading to the differential survival of the Equini.

## Supplemental Information

10.7717/peerj.2181/supp-1 Supplemental Information 1 Horse phylogeny

10.7717/peerj.2181/supp-2 Supplemental Information 2 Equid FADs and LADs

10.7717/peerj.2181/supp-3 Supplemental Information 3 Raw data used in this analysis

10.7717/peerj.2181/supp-4 Supplemental Information 4 R code used in analysis

## Institutional Abbreviations

AMNH F:AM: Frick Collection, American Museum of Natural History, New York City, New York, USA
MVZ: University of California Museum of Vertebrate Zoology, Berkeley, California, USA
UCMP: University of California Museum of Paleontology, Berkeley, California, USA
UF: University of Florida Museum of Natural History, Gainesville, Florida, USA
UNSM: University of Nebraska State Museum, Lincoln, Nebraska, USA
UOMNH: University of Oregon Museum of Natural and Cultural History, Eugene, Oregon, USA
USNM: United States National Museum of Natural History, Smithsonian Institute, Washington, District of Columbia, USA

## Additional Information and Declarations

### Data Availability

The following information was supplied regarding data availability: The raw data has been supplied as a Supplemental Dataset.
